# Association between apnea-hypopnea index and coronary artery calcification: a systematic review and meta-analysis

**DOI:** 10.1080/07853890.2021.1875137

**Published:** 2021-02-01

**Authors:** Wen Hao, Xiao Wang, Jingyao Fan, Yaping Zeng, Hui Ai, Shaoping Nie, Yongxiang Wei

**Affiliations:** a Emergency and Critical Care Center, Beijing Anzhen Hospital, Capital Medical University, Beijing, China; b Beijing Institute of Heart Lung and Blood Vessel Diseases, Beijing, China; c Department of Otolaryngology Head and Neck Surgery, Beijing Anzhen Hospital, Capital Medical University, Beijing, China

**Keywords:** Obstructive sleep apnoea, apnoea-hypopnea index, coronary artery calcification, coronary calcium score, subclinical coronary artery disease

## Abstract

**Background:**

The present study aimed to evaluate the association between presence and severity of obstructive sleep apnoea (OSA) and the presence of subclinical coronary artery disease (CAD) as assessed by coronary calcium score.

**Methods:**

Medline, Cochrane, and Google Scholar databases were searched. The presence of coronary artery calcification (CAC) and CAC score were assessed.

**Results:**

Irrespective of the cut-off value of apnoea-hypopnea index (AHI) (5 or 15 events/h), patients in the OSA group had higher rate of CAC presence and mean CAC score than those in the control group. Subgroup analyses of patients monitored with home sleep apnoea testing (HSAT) or in-hospital/laboratory polysomnography showed that the OSA group had higher rate of CAC presence and mean CAC score than the control group, except in the comparison of mean CAC score between AHI ≥5 vs. <5 events/h for patients using HSAT, which was not significant. Pair-wise comparison showed that CAC score may increase with increased OSA severity.

**Conclusions:**

In participants without symptomatic coronary disease, the presence of OSA was associated with the presence and extent of CAC. However, potential confounders such as age, gender, and BMI and the diversity of CAC scores may affect the association.

## Introduction

Obstructive sleep apnoea (OSA) is the most common type of sleep disordered breathing and is characterised by repeated episodes of complete or partial obstruction of the upper airway during sleep [1]. Approximately 1 billion adults aged between 30 and 69 years worldwide are estimated to exhibit OSA. According to the American Academy of Sleep Medicine, OSA syndrome is defined as an apnea-hypopnea index(AHI) ≥5 events/h with associated symptoms (e.g. excessive daytime sleepiness, impaired cognition, mood disorders, or insomnia, or documented hypertension, ischaemic heart disease, or history of stroke) or an AHI ≥15 events/h, regardless of associated symptoms [2]. The reported prevalence of OSA varied from 7.8% (Hong Kong) to 77.2% (Malaysia) with an AHI ≥5 events/h and from 4.8% (Ireland and Israel) to 36.6% (Switzerland) with an AHI ≥15 events/h [3]. The prevalence of OSA is disproportionately high in patients with cardiovascular disorders relative to the general population: hypertension (30–83%), ischaemic heart disease (30–58%), stroke (43–91%), heart failure (12–53%), and peripheral arterial disease (78–85%) [4–7]. Untreated OSA was associated with increased risk for cardiovascular morbidities, including stroke, hypertension, heart failure, and coronary artery disease (CAD), and increased risk of cardiovascular mortality [8]. Moreover, according to our previous research, OSA was associated with worse cardiovascular outcomes in patients with acute coronary syndrome in a cohort study as well as in patients who underwent percutaneous coronary intervention as revealed in a systematic review and meta-analysis [9,10]. According to findings of a systematic review and meta-analysis, the use of continuous positive airway pressure in patients with CAD and OSA may help prevent subsequent cardiovascular events [11].

Scoring coronary artery calcification (CAC) using computed tomography is a non-invasive assessment for individuals at risk for coronary atherosclerosis, and its use is becoming increasingly widespread [12]. CAC score was reported to be a strong predictor of CAD, and the risk of coronary events was increased with an increase in stratum of CAC score with the largest increase in risk associated with CAC scores >100 [13,14]. Although no randomised clinical trial has yet evaluated the effect of treatment according to CAC score, a considerable number of epidemiological and observational studies have provided convincing data worthy of consideration as guidance in clinical decision-making [12]. Furthermore, the use of CAC score to risk stratify asymptomatic patients has been considered appropriate/recommended by international guidelines; however, for symptomatic patients, the use of CAC score alone is limited [15].

An accumulating number of studies have investigated the link between OSA and subclinical coronary atherosclerosis assessed by CAC; however, the results have not been consistent. Some studies revealed that AHI was independently associated with CAC and that CAC score increased with OSA severity assessed by AHI, whereas other studies showed that the association was no longer significant after adjustment for possible confounders such as age, gender, and CAD risk factors [16–21]. The inconsistencies reflect the complex interactions between OSA, CAC, and traditional cardiovascular risk factors.

The purpose of the current systematic review and meta-analysis was to evaluate whether the presence of CAC and CAC score are associated with the presence of OSA using the AHI cut-off values of 5 and 15 events/h.

## Methods

### Search strategy

The study was conducted in accordance with the PRISMA guidelines [22]. Medline, Cochrane, and Google Scholar databases were searched up to April 29, 2020. The search terms used were: (obstructive sleep apnoea) OR (sleep characteristics) OR (sleep disturbance) OR (sleep disordered breathing); (coronary artery calcification) OR (coronary calcium) OR (coronary artery calcium); (subclinical coronary atherosclerosis) OR (subclinical coronary artery disease) OR (subclinical cardiovascular disease). The inclusion criteria were 1) clinical studies that included subjects with no known cardiovascular disease; 2) the severity of OSA was assessed by AHI; 3) the rate of CAC presence and/or CAC scores were reported; 4) English-language publications. Review articles, letters, books, commentaries, editorials, case reports, meeting proceedings, and personal communications were excluded. Studies designed for patients with end-stage renal disease or pulmonary fibrosis were also excluded.

A two-step process was used to screen the potential studies: (1) the title and abstract of each study was examined, and studies not meeting the inclusion criteria or meeting the exclusion criteria were discarded; (2) the full text of the remaining studies were examined for fulfilment of all inclusion criteria and none of the exclusion criteria. Two independent reviewers utilised the search strategy to identify eligible studies. A third reviewer was consulted if there was uncertainty regarding eligibility. The reference lists of relevant studies were searched manually to identify additional eligible studies.

### Data extraction

The following data were extracted from the included studies: name of the first author; year of publication; study design; number of participants in each group; participants’ age, gender and body mass index (BMI);comorbidities including hypertension, diabetes and dyslipidemia; polysomnographic findings including AHI, oxygen saturation and oxygen desaturation index; and major outcomes of CAC presence rate and CAC score.

### Quality assessment

The quality of the included studies was assessed using the 11 items suggested by the U.S. Agency for Healthcare Research and Quality (AHRQ) for assessing the quality of cross-sectional studies [23].

### Statistical analysis

The clinical characteristics were summarised as mean ± standard deviation (SD) or median (interquartile, IQR) for continuous variables, and n(%) for categorical ones. For the primary outcome, the presence of CAC defined as CAC score >0 was summarised as *n*(%) according toOSA severity classification. For the secondary outcome, CAC score was summarised as mean ± SD, mean (IQR), median (IQR), or median (range) according to OSA severity classification. Classification ofOSA severity was defined based on AHI:normal (AHI <5 events/h), mild (AHI ≥5 to <15 events/h), moderate (AHI ≥15 to <30 events/h), or severe (AHI ≥30 events/h) [2]. For meta-analysis, data were combined for two major comparisons: AHI ≥5 (OSA group) vs. <5 (control group) events/h and ≥15 (OSA group) vs. AHI <15 (control group) events/h. Moreover, the estimated sample mean and SD for CAC score were generated when data were presented with mean (IQR), median (IQR), or median (range) before proceeding with meta-analysis [24]. The measure of effect size for the presence of CAC was defined as odds ratio (OR) with 95%confidence interval (CI) and *p* value, and a combined effect was calculated thereby among those studies with complete measurements. An OR >1 indicated the rate of CAC presence was higher in the OSA group than in the control group, an OR <1 indicated the rate of CAC presence was lower in the OSA group than in the control group, and anORof1 indicated the rate of CAC presence was similar between groups.

The measure of effect size for CAC score was defined as difference in means of CAC score between groups with 95% CI and *p* value, and a combined effect was calculated thereby among those studies with complete measurements. For effect size, a difference in means >0 indicated the OSA group might have higher mean CAC score than the control group; a difference in means <0 indicated the OSA group might have lower mean CAC score than the control group; and a difference in means of 0 indicated CAC score was similar between groups.

Study heterogeneity was presented using a *χ*^2^-based Cochran’s *Q* statistic and *I*^2^statistic [25]. For the *Q* statistic, a corresponding *p* value of <.10 was considered statistically significant for heterogeneity. For the *I*^2^ statistic, heterogeneity was assessed as follows: no heterogeneity (*I*^2^ = 0–25%), moderate heterogeneity (*I*^2^ = 25–50%), large heterogeneity (*I*^2^ = 50–75%), and extreme heterogeneity (*I*^2^= 75–100%). If *p* value was <0.10 for *χ*^2^-based Cochran’s Q statistic or if *I*^2^>50%, a random-effect model was considered to estimate the pooled effect; otherwise, a fixed-effect model was performed [26]. A two-sided *p* value of <.05 was considered significant. A sensitivity analysis was conducted using a leave-one-out approach. Publication bias was not assessed due to the small number of included studies [27]. A subgroup analysis was performed to calculate the pooled effect in patients monitored with home sleep apnoea testing (HSAT) or in-hospital/laboratory polysomnography (PSG). Moreover, pairwise comparisons among OSA severity groups were performed to evaluate the association of the presence of CAC and CAC score with OSA severity. All statistical analyses were performed using the statistical software Comprehensive Meta-Analysis, version 2.0 (Biostat, Englewood, NJ).

## Results

### Search results

**Figure 1.**
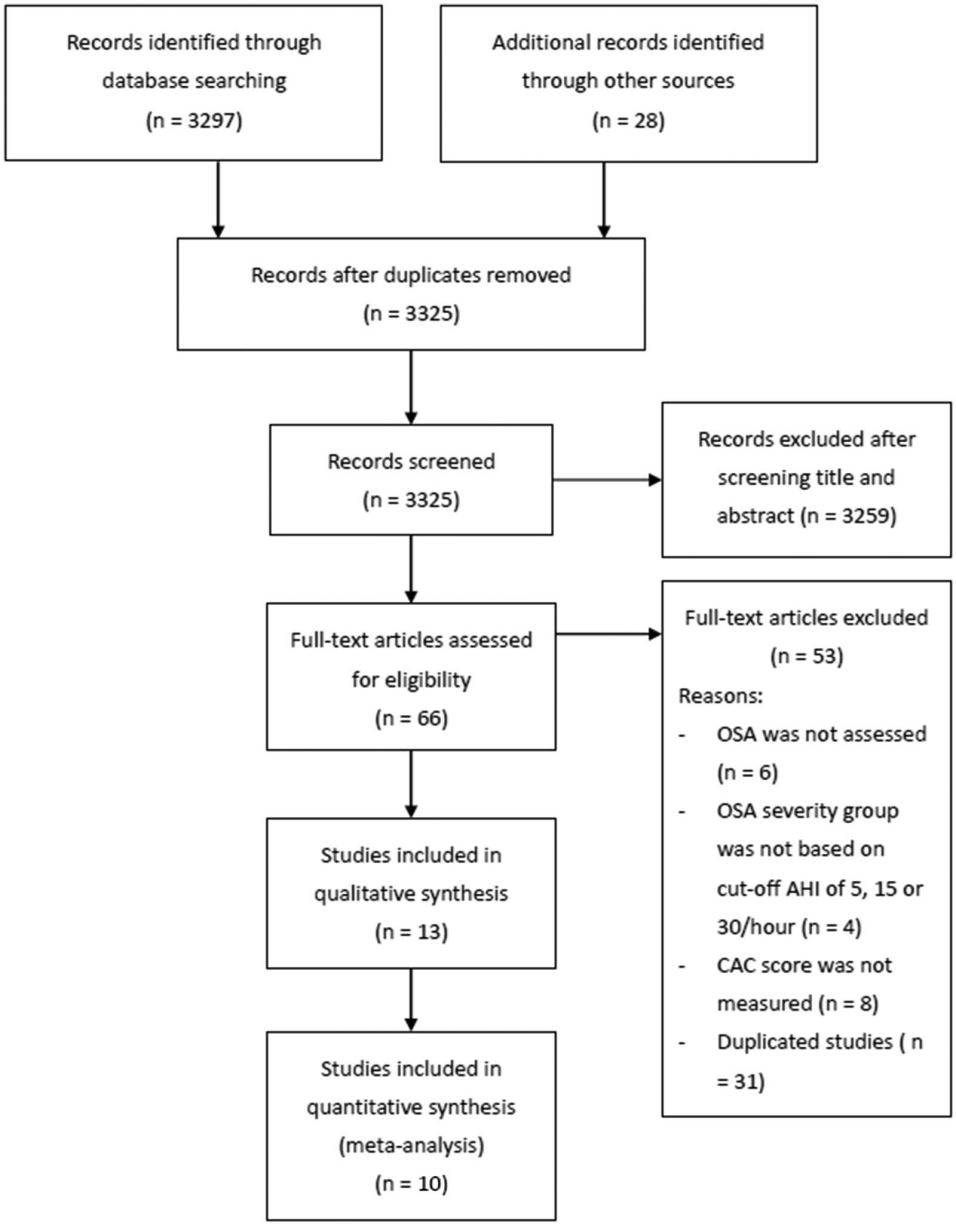
Flow diagram of study selection.

The flow diagram of study selection is shown in Figure 1. A total of 3325 articles were initially screened using titles and abstracts, of which 3259 were excluded. After full-text review of the remaining of 66 articles, 53 studies were excluded, and the reasons for exclusion are shown in Figure 1. Therefore, 13 articles were included in the systematic review [16–20,28–35]. Subjects in Matthews *et al.* [35], Luyster *et al.* [34], and Shipilsky *et al.* [30] were recruited from the Heart Strategies Concentrating on Risk Evaluation (HeartSCORE) study; among these, Shipilsky *et al.* [30] was the only study not included in the meta-analysis. Seo *et al.* [19] and Hamaoka *et al.* [31] reported data in a form that could not be pooled with data from the other studies. Ultimately, 10 studies were included in the meta-analysis.

### Characteristics of included studies

The characteristics of the included studies are summarised in Table 1. 12 studies were cross-sectional in design. Overall, 6620 subjects were included, with mean age ranging from 46 to 69.1 years and proportion of males ranging from 0 to 92.8%. Mean BMI ranged from 23.8to 35 kg/m^2^. Reported comorbidities and polysomnographic recordings are also summarized in Table 1. Overall, the percentage of patients with hypertension ranged from 21 to 95%. The percentage of patients with diabetes ranged from 4 to 43.2%. Included inthe10studiescomprising the meta-analysis were 2,450 patients without OSA (AHI<5) and 3,677 patients with mild to severe OSA (AHI ≥5). In terms of OSA severity,4,406 patients had no to mild OSA (AHI< 15) and 1,456 patients had moderate to severe OSA (AHI ≥15). Table 2 summarizes the rate of CAC presence and CAC score in each OSA severity group.

**Table 1.** Demographic and clinical characteristics for enrolled studies.

First author (published year)	Study design	Trial name	Country	Ethnicity	OSA severity	Assessment of OSA	Number of patients	Age (Mean ± SD years)	Sex, Males (%)	BMI (Mean ± SD kg/m^2^)
Kim (2020)	CS	KoGES	Korea	–	Normal	HSAT	1096	58.8 ± 6.0	456 (42)	23.8 ± 2.6
					Mild		700	60.8 ± 6.8	349 (50)	25.2 ± 2.9
					Moderate to Severe		361	62.1 ± 7.6	246 (68)	26.1 ± 3.3
Bikov (2019)	CS	part of participants were selected from the BUDAPEST‐GLOBAL	Hungary	–	Normal	In‐hospital PSG	19	56 ± 9	3 (16)	26.3 ± 3.8
					Mild, Moderate to Severe		44	62 ± 10	24 (55)	29.4 ± 5.7
Shipilsky (2018)	CS	Heart SCORE	USA	black (37%)	Normal/Mild	HSAT (ApneaLink)	561	58 ± 7	159 (28)	30 ± 6
					Moderate to Severe		204	61 ± 7	108 (53)	31 ± 6
Hamaoka (2018)	CS		Japan	–	Mild to Moderate	In-laboratory PSG	15	61.3 ± 12.4	11 (73.3)	26.3 ± 5.0
					Severe		17	65.0 ± 9.2	15 (88.2)	26.2 ± 5.3
Seo (2017)	CS		Korea	–	Normal	In‐hospital PSG	64	55.08 ± 7.65	428 (92.8)	25.57 ± 3.01
					Mild		121			
					Moderate		123			
					Severe		153			
Medeiros (2017)	CS		Brazil	–	Normal	In-laboratory PSG	132	55 (51–59)^#^	0 (0)	27(24–29)^#^
					Mild		61	59 (54–62)^#^		29 (25–34)^#^
					Moderate to Severe		21	58 (53–63)^#^		32 (29–35)^#^
Lutsey (2015)	CS	MESA	USA	non-Hispanic African American (28%), Chinese (12%), Caucasian (38%) or Hispanic (22%)	Normal	HSAT	510	66.9 ± 9.0	165 (32)	26.6 ± 5.0
					Mild		478	68.9 ± 9.2	214 (45)	28.9 ± 5.0
					Moderate		263	69.1 ± 9.2	155 (59)	29.6 ± 5.3
					Severe		214	67.9 ± 9.0	136 (64)	31.7 ± 6.0
Luyster (2014)	CS	Heart SCORE	USA	European American: 156 (62%); African American: 96 (38%)	Normal	HSAT (ApneaLink)	61	59.1 ± 7.8	29 (48)	29.0 ± 5.8
					Mild		97	60.9 ± 7.6	48 (49)	29.9 ± 4.5
					Moderate to Severe		94	62.0 ± 6.5	64 (68)	30.4 ± 5.0
Arik (2013)	Pro		Turkey	–	Normal	In‐hospital PSG	16	50 ± 10	9 (56)	28.2 ± 4.8
					Mild		14	48 ± 9	6 (43)	30.9 ± 6.4
					Moderate		19	47 ± 9	13 (68)	28.4 ± 4.8
					Severe		24	52 ± 10	15 (63)	32.2 ± 4.1
Weinreich (2013)	CS	Nixdorf Recall	German	–	Normal (M)	HSAT (ApneaLink)	209	61.9 ± 6.9	209 (100)	27.6 ± 3.6
					Mild (M)		327	63.4 ± 7.4	327 (100)	28.4 ± 3.5
					Moderate (M)		176	65.1 ± 7.3	176 (100)	28.6 ± 3.7
					Severe (M)		79	65.9 ± 7.7	79 (100)	30.2 ± 3.9
					Normal (F)		342	60.8 ± 6.8	0 (0)	26.2 ± 4.2
					Mild (F)		324	64.6 ± 7.2	0 (0)	27.7 ± 4.6
					Moderate (F)		112	66.1 ± 7.4	0 (0)	28.8 ± 5.1
					Severe (F)		35	68.5 ± 5.7	0 (0)	30.9 ± 4.1
Matthews (2011)	CS	HeartSCORE ≥ SleepSCORE		African American: 97; Caucasian: 123; Asian: 4	Normal	HSAT	63	N/A	113 (57)	
					Mild to Moderate		134	N/A		
					Severe		24			
Kepez (2011)	CS		Turkey	–	Normal	In‐hospital PSG	17	46.60 ± 4.59	10 (58.8)	28.25 ± 4.04
					Mild		22	46.56 ± 9.54	16 (72.7)	27.61 ± 3.09
					Moderate		21	52.17 ± 7.20	16 (76.2)	29.18 ± 3.28
					Severe		37	49.85 ± 10.60	22 (59.5)	30.99 ± 4.43
Sorajja (2008)	CS		USA	–	Normal	In‐hospital PSG	48	mean = 46	30 (63)	mean = 31
					Mild, Moderate to Severe		154	mean = 51	111 (72)	mean = 35

OSA severity group was defined according to apnoea-hypopnea index (AHI): normal (AHI <5 events/h), mild (AHI ≥ 5 to < 15 events/h), moderate (AHI ≥15 to <30 events/h), or severe (AHI ≥30 events/h).

^#^median (IQR).

CS: cross-sectional study; F: female; HSAT: home sleep apnoea testing; M: male; OSA: obstructive sleep apnoea; Pro: prospective study; PSG: polysomnography.

**Table 1.** (Continued).

First author (published year)	OSA severity	Hypertension, *n* (%)	Diabetes, *n* (%)	Dyslipidemia *n* (%)	AHI, Mean ± SD event/h	Mean SaO_2_, Mean ± SD %	Lowest SaO_2_, Mean ± SD %	Oxygen desaturation index, Mean ± SD event/h
Kim (2020)	Normal	374 (34.1)	281 (25.6)	N/A	2.1 ± 1.4	95.9 ± 1.1	89.6 ± 5.3	1.9 ± 0.3
	Mild	353 (50.4)	227 (32.4)	N/A	8.7 ± 2.7	95.2 ± 1.2	85.1 ± 4.7	7.9 ± 2.9
	Moderate to Severe	226 (62.6)	156 (43.2)	N/A	26.1 ± 12.1	94.4 ± 1.5	80.4 ± 6.7	24.0 ± 11.6
Bikov (2019)	Normal	12 (63)	3 (16)	10 (53)	1.8 ± 1.1	N/A	89.8 ± 3.1	1.6 ± 1.1
	Mild, Moderate to Severe	34 (77)	8 (18)	22 (50)	18.8 ± 16.3	N/A	83.9 ± 3.9	15.8 ± 15.9
Shipilsky (2018)	Normal/Mild	199 (36)	43 (8)	N/A	5.9 ± 3.8	N/A	N/A	N/A
	Moderate to Severe	104 (51)	22 (11)	N/A	26.1 ± 12.0	N/A	N/A	N/A
Hamaoka (2018)	Mild to Moderate	5 (30.0)	2 (13.3)	3 (20.0)	20.7 ± 8.4	N/A	N/A	N/A
	Severe	9 (52.9)	4 (23.5)	5 (29.4)	45.2 ± 11.9	N/A	N/A	N/A
Seo (2017)	Normal	205 (44.5)	62 (13.4)	268 (58.1)	25.46 ± 21.10	N/A	83.56 ± 7.03	N/A
	Mild							
	Moderate							
	Severe							
Medeiros (2017)	Normal	92 (70)	32 (24)	94 (77)	1.9 (0.5–3.3)^#^	96 (96–98)^#^	91 (89–93)^#^	N/A
	Mild	50 (82)	15 (25)	45 (75)	8.3 (6.0–11.1)^#^	96 (95–96)^#^	85 (82–88)^#^	N/A
	Moderate to Severe	20 (95)	6 (29)	14 (70)	16.9 (15.4–24.5)^#^	95 (94–96)^#^	80 (75–84)^#^	N/A
Lutsey (2015)	Normal	N/A	71 (14.0)	N/A	N/A	N/A	N/A	N/A
	Mild	N/A	90 (19.0)	N/A	N/A	N/A	N/A	N/A
	Moderate	N/A	63 (24.1)	N/A	N/A	N/A	N/A	N/A
	Severe	N/A	51 (23.8)	N/A	N/A	N/A	N/A	N/A
Luyster (2014)	Normal	43 (71)	10 (16)	45 (77)	2.7 ± 1.1	N/A	N/A	N/A
	Mild	65 (67)	22 (23)	81 (83)	9.0 ± 2.7	N/A	N/A	N/A
	Moderate to Severe	71 (75)	19 (20)	65 (69)	26.9 ± 12.3	N/A	N/A	N/A
Arik (2013)	Normal	6 (38)	4 (25)	6 (38)	2.4 ± 1.1	N/A	94 ± 6	0.7 ± 2.7
	Mild	5 (36)	2 (14)	3 (21)	8.0 ± 2.0	N/A	87 ± 10	2.7 ± 3.2
	Moderate	4 (21)	6 (32)	3 (16)	22.5 ± 4.3	N/A	77 ± 12	5.1 ± 3.3
	Severe	12 (50)	9 (38)	9 (38)	45.3 ± 16.3	N/A	71 ± 12	14.6 ± 19.7
Weinreich (2013)	Normal (M)	123 (58.9)	27 (12.9)	N/A	N/A	N/A	N/A	N/A
	Mild (M)	230 (70.3)	42 (12.8)	N/A	N/A	N/A	N/A	N/A
	Moderate (M)	125 (71)	26 (14.7)	N/A	N/A	N/A	N/A	N/A
	Severe (M)	63 (79.8)	8 (10.10)	N/A	N/A	N/A	N/A	N/A
	Normal (F)	149 (43.6)	30 (8.8)	N/A	N/A	N/A	N/A	N/A
	Mild (F)	190 (58.6)	30 (9.3)	N/A	N/A	N/A	N/A	N/A
	Moderate (F)	69 (61.6)	13 (11.6)	N/A	N/A	N/A	N/A	N/A
	Severe (F)	25 (71.4)	5 (14.3)	N/A	N/A	N/A	N/A	N/A
Matthews (2011)	Normal	94 (42.0)	N/A	N/A	N/A	N/A	N/A	N/A
	Mild to Moderate		N/A	N/A	N/A	N/A	N/A	N/A
	Severe		N/A	N/A	N/A	N/A	N/A	N/A
Kepez (2011)	Normal	4 (23.5)	2 (11.8)	2 (12.5)	N/A	94.30 ± 1.20	94.34 ± 1.32	0.00 (1.00)^#^
	Mild	7 (31.8)	2 (9.1)	6 (27.3)	N/A	94.12 ± 1.63	94.45 ± 1.53	2.00 (2.25)^#^
	Moderate	7 (33.3)	2 (9.5)	5 (23.8)	N/A	93.04 ± 1.20	93.58 ± 1.41	4.00 (7.00)^#^
	Severe	15 (40.5)	7 (18.9)	11 (29.7)	N/A	91.20 ± 3.40	92.38 ± 2.48	13.00 (24.75)^#^
Sorajja (2008)	Normal	20 (40)	2 (4)	23 (48)	N/A	N/A	N/A	N/A
	Mild, Moderate to Severe	76 (49)	14 (9)	99 (64)	N/A	N/A	N/A	N/A

OSA severity group was defined according to apnoea-hypopnea index (AHI): normal (AHI < 5 events/h), mild (AHI ≥ 5 to < 15 events/h), moderate (AHI ≥ 15 to < 30 events/h), or severe (AHI ≥ 30 events/h).

AHI: apnoea-hypopnea index; BMI: body mass index; F: female; M: male; SaO_2_: oxygen saturation; NA: not available; OSA: obstructive sleep apnoea.

^#^median (IQR).

**Table 2.** Summary of primary and secondary outcomes based on OSA severity.

First author (published year)	OSA severity	Number of patients	CAC score	Presence of CAC, *n* (%)	Definition of CAC
Kim (2020)	Normal	1096	0.0 (0.0–3039.4)^†^	248 (22.63)	CAC score > 0
	Mild	700	0.0 (0.0–1583.4)^†^	181 (25.86)	
	Moderate to Severe	361	0.0 (0.0–1808.6)^†^	131 (36.39)	
Bikov (2019)	Normal	19	20.9 ± 67.6	4 (21)	CAC score > 0
	Mild, Moderate to Severe	44	137.4 ± 218.4	20 (45)	
Shipilsky (2018)	Normal/Mild	561	17 (0–107)*	N/A	CAC scor*e* > 0 was considered positive. CAC scor*e* > 100 corresponds to significant CAC burden
	Moderate to Severe	204	Moderate: 44 (8–143)* severe: 101 (11–321)*	N/A	
Hamaoka (2018)	Mild to Moderate	15	120.3 ± 307.8	N/A	A calcific lesion was defined as an are*a* ≥ 1 mm^2^ above 130 Hounsfield Units.
	Severe	17	281.0 ± 275.6	N/A	
Seo (2017)	Normal	64	89.4 ± 300.54	272 (59%)	The presence of CAC was considered when the CT density was 130 Hounsfield units having an area 1mm^2^.
	Mild	121			
	Moderate	123			
	Severe	153			
Medeiros (2017)	Normal	132	N/A	CA*C* > 100: 6 (4.5)	CAC scor*e* > 100 were considered with coronary atherosclerosis
	Mild	61	N/A	CA*C* > 100: 1 (1.6)	
	Moderate to Severe	21	N/A	CA*C* > 100: 4 (19)	
Lutsey (2015)	Normal	510	median 6.2	280 (54.9)	CAC scor*e* > 0 was considered prevalent. CAC scor*e* > 400 was considered high CAC burden.
	Mild	478	median 32.6	318 (66.5)	
	Moderate	263	median 31.8	177 (67.3)	
	Severe	214	median 62.9	16 (75.7)	
Luyster (2014)	Normal	61	18.3 (0–786)^†^	41 (67.2)	CAC score > 0
	Mild	97	18.1 (0–1359)^†^	70 (72.2)	
	Moderate to Severe	94	64.2 (0–1115)^†^	79 (84)	
Arik (2013)	Normal	16	1.4 ± 3.0 (range: 0–11)	4 (25)	CAC score > 0
	Mild	14	1.9 ± 4.9 (rang: 0–15)	2 (14)	
	Moderate	19	32.2 ± 97.5 (range: 0–420)	7 (37)	
	Severe	24	59.5 ± 121.2 (range: 0–480)	14 (58)	
Weinreich (2013)	Normal (M)	209	49 [0–311]^#^	N/A	CAC was defined as a focus of at least 4 contiguous pixels with a CT density 130 Hounsfield Units.
	Mild (M)	327	83 [7–381]^#^	N/A	
	Moderate (M)	176	134 [21–444]^#^	N/A	
	Severe (M)	79	165 [31–439]^#^	N/A	
	Normal (F)	342	0 [0–26]^#^	N/A	
	Mild (F)	324	2 [0–55]^#^	N/A	
	Moderate (F)	112	8 [0–208]^#^	N/A	
	Severe (F)	35	40 [0–354]^#^	N/A	
Matthews (2011)	Normal	63	115.1 ± 269.1 (range: 0.0–1519.1	29 (53.7)	CAC score > 0
	Mild to Moderate	134		87 (72.5)	
	Severe	24		15 (78.9)	
Kepez (2011)	Normal	17	4.61 ± 13.29	N/A	
	Mild	22	58.23 ± 175.23	N/A	
	Moderate	21	32.40 ± 63.46	N/A	
	Severe	37	53.22 ± 196.24	N/A	
Sorajja (2008)	Normal	48	median: 0; mean: 26	15 (31)	Patients were classified as having subclinical coronary disease if the CAC score wa*s* > 0.
	Mild, Moderate to Severe	154	median: 9; mean: 144	103 (67)	

OSA severity group was defined according to apnoea-hypopnea index (AHI): normal (AHI <5 events/h), mild (AHI ≥5 to <15 events/h), moderate (AHI ≥15 to <30 events/h), or severe (AHI ≥30 events/h).

CAC: coronary artery calcification; F: female; M: male; NA: not available; OSA: obstructive sleep apnoea.

CAC score was represented as Mean ± SD.

*mean (IQR); ^†^median (range); ^#^median (IQR).

### Meta-analysis

#### Association of OSA severity and presence of CAC

**Figure 2.**
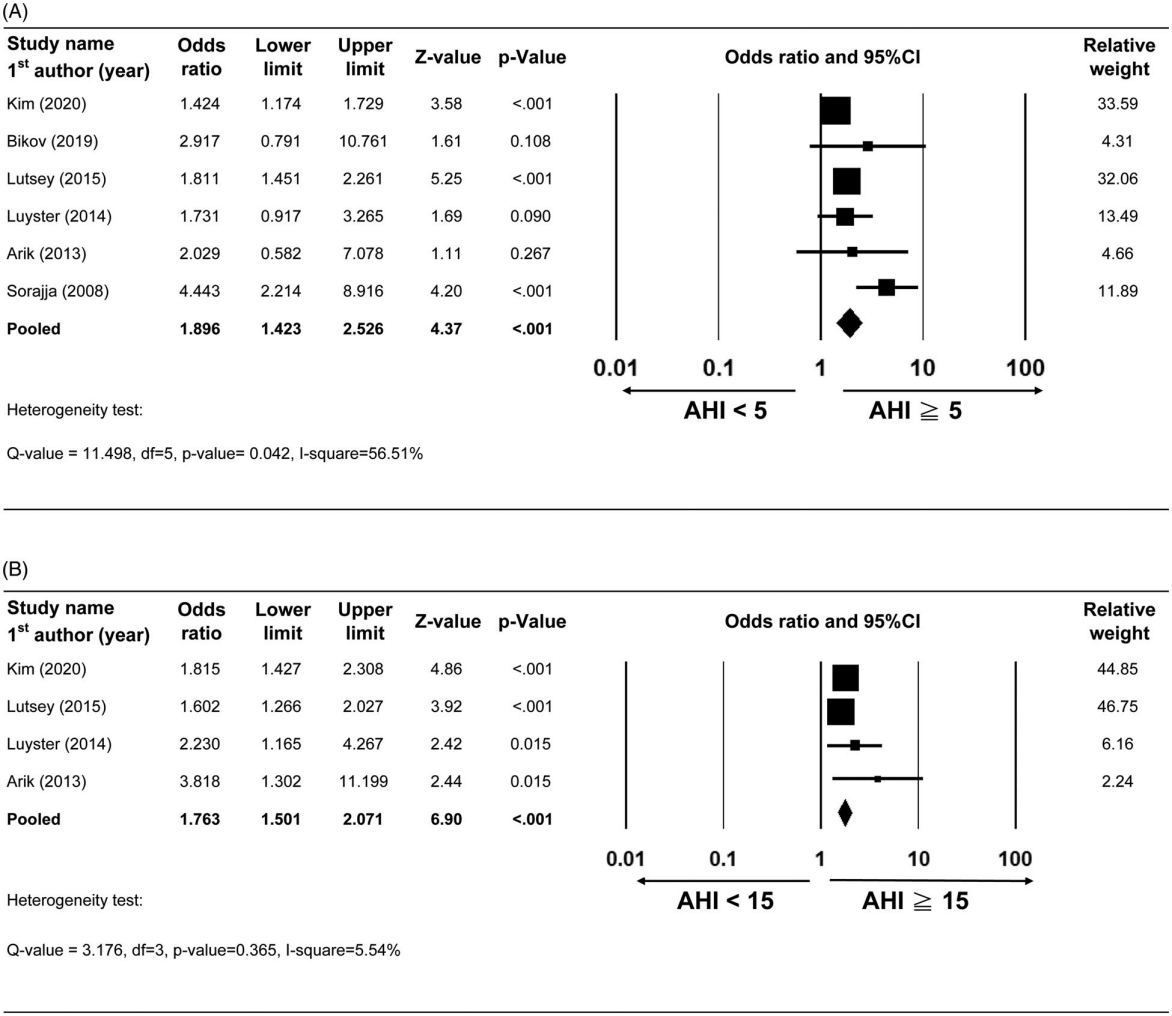
Forest plot comparing presence of CAC between (A) AHI ≥5 vs.<5events/h and (B) AHI ≥15 vs.<15events/h. Abbreviations: 95% CI, 95% confidence interval of odds ratio (lower limit and upper limit).

Six studies with complete data on CAC presenc efor the comparison between AHI ≥5 (OSA group) and AHI <5 (control group) events/h were included for meta-analysis [16,18,28,29,33,34]. The rate of CAC presence ranged from 21 to 67.2% in the control group and from 29.4 to 78% in the OSA group. A random-effect model was applied according to the results of heterogeneity tests (Q-value = 11.50, df = 5, *p* value =.042, *I*^2^ = 56.51%). The combined effect revealed that patients in the OSA group might have a higher rate of CAC presence compared with those in the control group (pooled OR = 1.896, 95%CI = 1.423–2.526, *p* value < .001) (Figure 2(A)).

Four studies with complete data on the presence of CAC for the comparison of AHI ≥15 (OSA group) and AHI <15 (control group) events/h were included for meta-analysis [16,28,33,34]. The rate of CAC presence ranged from 20 to 70.2% in the control group and from 36.3 to 84.0% in the OSA group. A fixed-effect model was applied according to results of heterogeneity tests (Q-value = 3.17, df = 3, *p* value = .365, I-square = 5.54%). The combined effect revealed that patients in the OSA group might have a higher rate of CAC presence compared with those in the control group(pooled OR = 1.763, 95%CI = 1.501 to 2.071, *p* value < .001) (Figure 2(B)).

Results of the subgroup analyses showed that regardless of the cut-off value of AHI (5 or 15 events/h) used, the OSA group might have a higher rate of CAC presence than the control group in patients monitored with either HSAT or in-hospital/laboratory PSG (all ORs >1; *p* values < .001). The pooled result of in-hospital/laboratory PSG with regard to AHI ≥15 vs. <15 events/h was not assessed because only one study reported such data. Pair-wise comparison showed that the association between the presence of CAC and OSA severity was significant in patients with mild and moderate OSA compared to patients without OSA (all ORs >1; *p* values ≤.001). However, the significance was not observed in patients with moderate-to-severe and severe OSA compared to patients without OSA (Table 3).

**Table 3.** Subgroup analysis and pair-wise comparison of presence of CAC and CAC score.

Comparisons	Pooled statistics	Number of studies	Heterogeneity test
Presence of CAC			
Subgroup analysis			
HSAT			
AH*I* ≥ 5 vs. < 5	O*R* = 1.59, 95%CI=(1.38, 1.83), *p* < .001	3	Q-valu*e* = 2.633, *p* value = .268, *I*^2^=24.03%
AH*I* ≥ 15 vs. < 15	O*R* = 1.73, 95%CI=(1.47, 2.04), *p* < .001	3	Q-valu*e* = 1.149, *p* value = .563, *I*^2^=0%
In-hospital/laboratory PSG			
AH*I* ≥ 5 vs. < 5	O*R* = 3.54, 95%CI=(2.04, 6.14), *p* < .001	3	Q-valu*e* = 1.255, *p* value = .534, *I*^2^=0%
Pair-wise Comparison			
Mild vs. normal	O*R* = 1.35, 95%CI=(1.14, 1.58), *p* < .001	4	Q-valu*e* = 4.411, *p* value = .220, *I*^2^=31.98%
Moderate vs. normal	O*R* = 1.69, 95%CI=(1.25, 2.29), *p* = 0.001	2	Q-valu*e* = 0.002, *p* value = .964, *I*^2^=0%
Severe vs. normal	O*R* = 0.81, 95%CI=(0.05, 12.35, *p* = 0.879	3	Q-valu*e* = 84.315, *p* value < .001, *I*^2^=97.63%
Moderate to severe vs. normal	O*R* = 1.56, 95%CI=(0.64, 3.81), *p* = 0.329	4	Q-valu*e* = 53.489, *p* value <.001, *I*^2^=94.39%
CAC score			
Subgroup analysis			
HSAT			
AH*I* ≥ 5 vs. < 5	diff in mean*s* = 26.13, 95%CI=(−6.67, 58.93), *p* = .118	3	Q-valu*e* = 6.558, *p* value = .038, *I*^2^=69.50%
AH*I* ≥ 15 vs. < 15	diff in mean*s* = 42.39, 95%CI=(2.64, 82.15), *p* = .037	3	Q-valu*e* = 7.099, *p* value = .029, *I*^2^=71.83%
In-hospital/laboratory PSG			
AH*I* ≥ 5 vs. < 5	diff in mean*s* = 51.39, 95%CI=(9.67, 93.11), *p* = .016	3	Q-valu*e* = 1.949, *p* value = .344, *I*^2^ = 0%
AH*I* ≥ 15 vs. < 15	diff in mean*s* = 35.25, 95%CI=(1.06, 69.44), *p* = .043	2	Q-valu*e* = 0.848, *p* value = 0.357, *I*^2^=0%
Pair-wise Comparison			
Mild vs. normal	diff in mean*s* = 11.633, 95%CI=(−7.795, 31.060), *p* = .241	5	Q-valu*e* = 43.991, *p* value < .001, *I*^2^=90.91%
Moderate vs. normal	diff in mean*s* = 46.194, 95%CI=(15.708, 76.679), *p* = .003	3	Q-valu*e* = 7.324, *p* value = .026, *I*^2^=72.69%
Severe vs. normal	diff in mean*s* = 86.211, 95%CI=(45.152, 127.269), *p* < .001	3	Q-valu*e* = 4.071, *p* value = .131, *I*^2^=50.871%
Moderate to severe vs. normal	diff in mean*s* = 45.512, 95%CI=(15.351, 81.672), *p* = .004	5	Q-valu*e* = 10.266, *p* value = .036, *I*^2^=61.04%

OSA severity group was defined according to apnoea-hypopnea index (AHI): normal (AHI <5 events/h), mild (AHI ≥5 to <15 events/h), moderate (AHI ≥15 to <30 events/h), or severe (AHI ≥30 events/h).

CAC: coronary artery calcification; diff: difference; HSAT: home sleep apnoea testing; PSG: polysomnography; OR: odds ratio.

#### Association of OSA severity and CAC score

**Figure 3.**
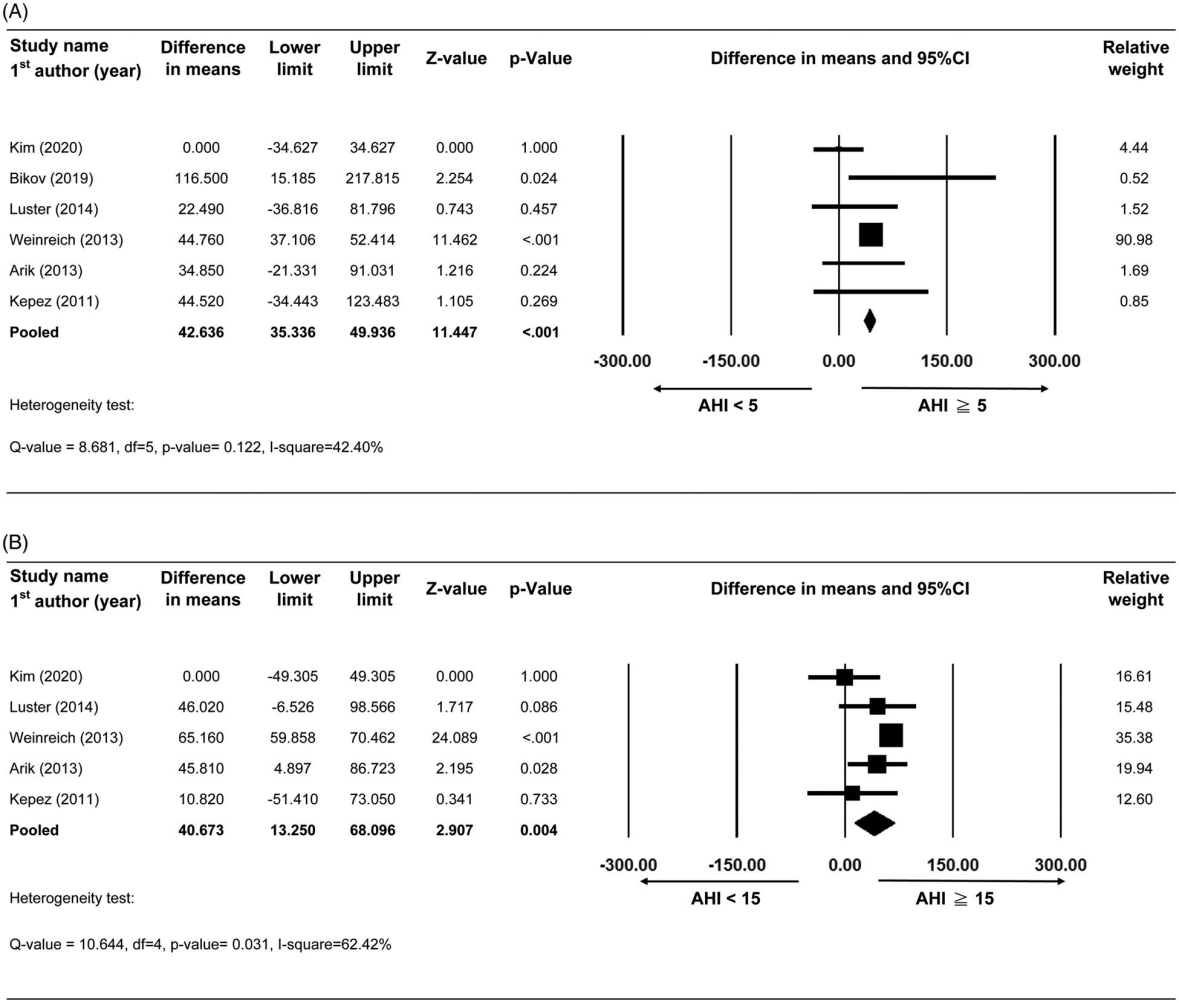
Forest plot comparing CAC scores between (A) AHI ≥5 vs.<5events/h and (B) AHI ≥15 vs.<15events/h. Abbreviations: 95% CI, 95% confidence interval of difference in means (lower limit and upper limit).

Six studies with complete data on CAC score for the comparison of AHI ≥5(OSA group) and AHI <5 (control group)events/h were included for meta-analysis [16,17,20,28,29,34]. Mean CAC scores ranged from 0 to 20.9 in the control group and from 0 to 137.4 in the OSA group. A fixed-effect model was applied according to results of heterogeneity tests (Q-value = 8.681, df = 5, *p* value=.122, I-square = 42.40%). The combined effect showed that the mean CAC score was higher in OSA group than in control group (difference in means of CAC score = 42.64, 95%CI = 35.34 to 49.94, *p* value < .001) (Figure 3(A)).

Five studies with complete data on CAC score for the comparison of AHI ≥15(OSA group) and AHI <15 (control group) events/h were included for meta-analysis [16,17,20,28,34]. Mean CAC scores ranged from 0 to 34.8 in the control group and from 0 to 96.8 in the OSA group. A random-effect model was applied according to results of heterogeneity tests (Q-value = 10.644, df = 4, *p* value =.031, I-square = 62.42%). The combined effect showed that mean CAC score was higher in the OSA group than in the control group (difference in means of CAC score = 40.67, 95%CI = 13.25 to 68.10, *p* value = .004) (Figure 3(B)).

Results of the subgroup analyses suggest that the OSA group might have higher mean CAC score than the control group in patients monitored with either HSAT or in-hospital/laboratory PSG (all pooled effect, differences in means >0); however, the significance was not observed in the comparison of AHI ≥5 vs. <5 events/h in patients using HSAT. Pair-wise comparison showed that the pooled effect was significantly different for comparisons between moderate, severe, and moderate-to-severe OSA vs. no OSA (differences in means = 46.19, 86.21, and 45.51; *p* = .003, <0.001, and 0.004, respectively); however, there was no significant difference between mild OSA and no OSA (difference in means = 11.63, *p* = .241) (Table 3).

#### Sensitivity analysis

**Figure 4.**
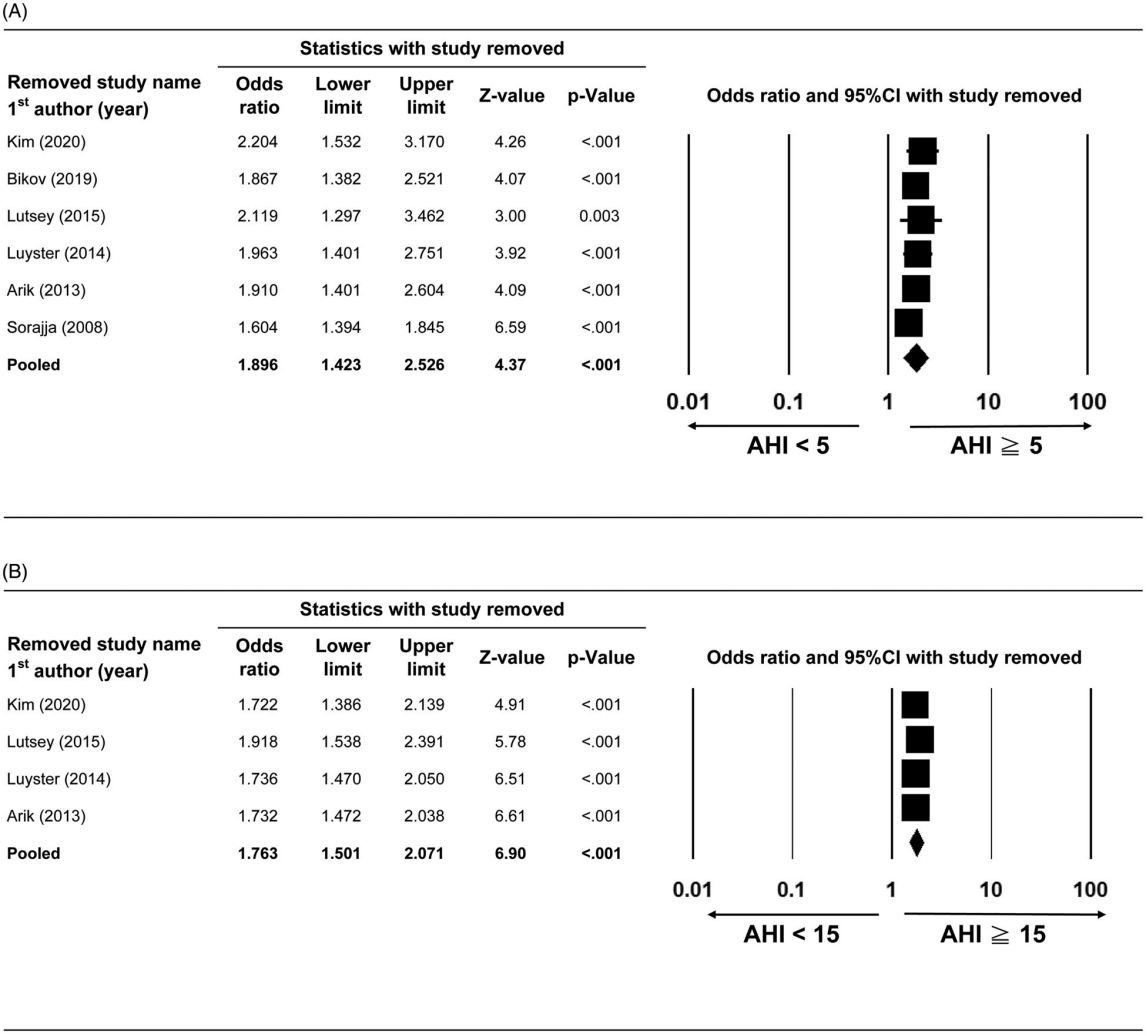
Sensitivity analyses of presence of CAC between (A) AHI ≥5 vs.<5 events/h and (B) AHI ≥15 vs.<15events/h. Abbreviations: 95% CI, 95% confidence interval of odds ratio (lower limit and upper limit).

**Figure 5.**
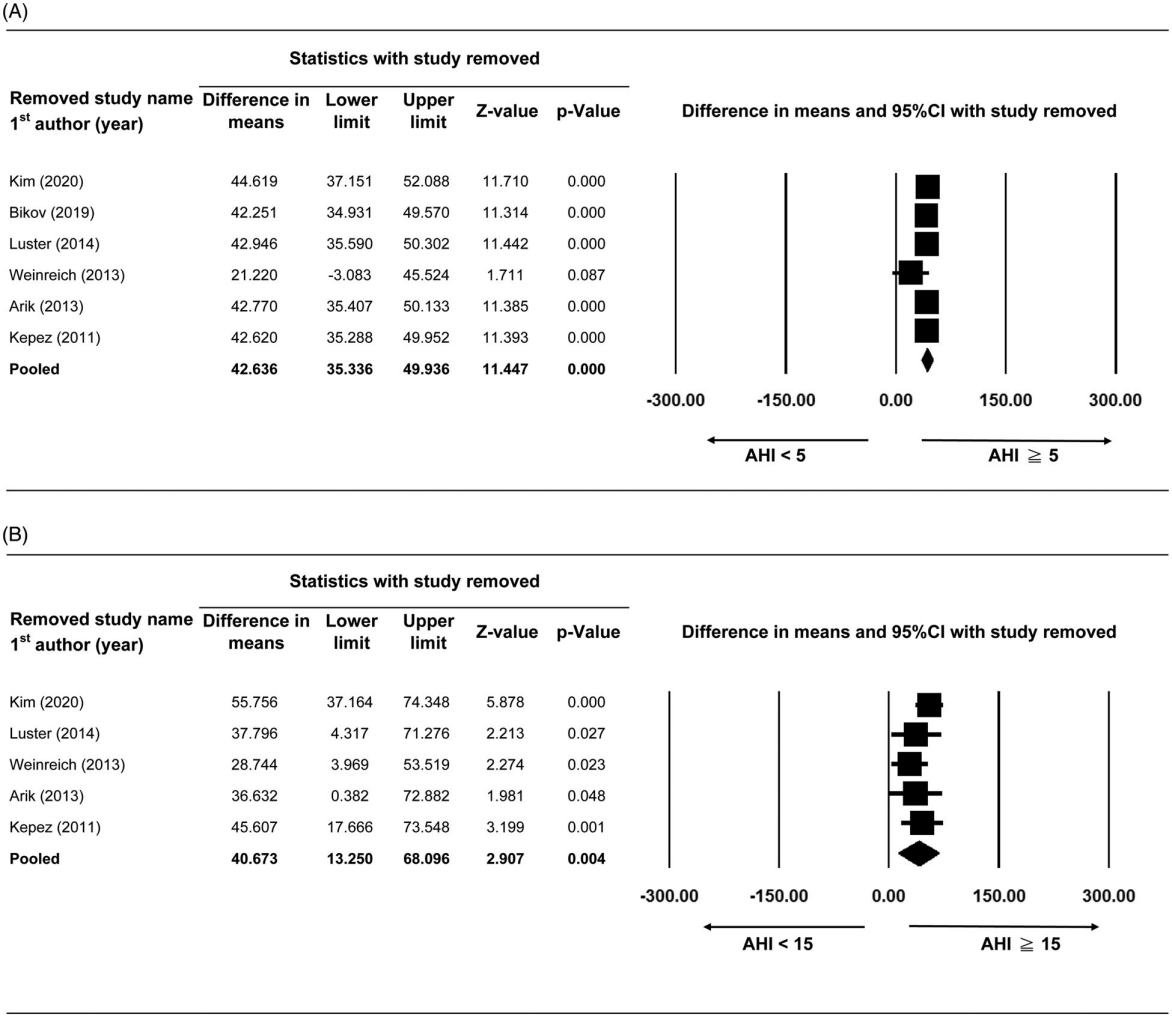
Sensitivity analysis of CAC score between (A) AHI ≥5 vs.<5 events/h and (B) AHI ≥15 vs.<15events/h. Abbreviations: 95% CI, 95% confidence interval of difference in means (lower limit and upper limit.

Sensitivity analyses were performed for the primary and secondary outcomes using a leave-one-out approach. The direction and magnitude of the combined estimates for the presence of CAC (Figure 4) and CAC score in AHI <15 vs. AHI ≥15 events/h (Figure 5(B)) did not markedly differ with the removal of any one study, indicating that the meta-analysis had good reliability and that the data was not overly influenced by any given study. Pooled difference in means of CAC score in AHI ≥5 vs. AHI <5events/h was still >0 despite that the difference in means of CAC score became borderline significant after the removal of Weinreich *et al.* [17], indicating no obvious influence on the pooled estimate (Figure 5(A)).

#### Quality assessment

The quality of the included studies was evaluated using the ARHQ methodology checklist, and the results are shown in Table 4. All of the included studies reported the source of data, patient inclusion and exclusion criteria, and summarised patient response; however, the completeness of data collection was unclear. Most of the included studies indicated whether participants were consecutive and how confounding was assessed.

**Table 4.** Quality assessment of included studies.

First author (published year)	Kim (2020)	Bikov (2019)	Shipilsky (2018)	Hamaoka (2018)	Seo (2017)	Medeiros (2017)	Lutsey (2015)	Lutsey (2014)	Arik (2013)	Weinreich (2013)	Matthews (2011)	Kepez (2011)	Sorajja (2008)
Define the source of information (survey, record review)	Yes	Yes	Yes	Yes	Yes	Yes	Yes	Yes	Yes	Yes	Yes	Yes	Yes
List inclusion and exclusion criteria for exposed and unexposed participants (cases and controls) or refer to previous publications	Yes	Yes	Yes	Yes	Yes	Yes	Yes	Yes	Yes	Yes	Yes	Yes	Yes
Indicate time period used for identifying patients	Yes	No	No	Yes	Yes	Yes	Yes	No	No	Yes	No	No	Yes
Indicate whether or not Participants were consecutive if not population based	Yes	Yes	Yes	No	No	Yes	No	Yes	Yes	Yes	Yes	Yes	Yes
Indicate whether evaluators of subjective components of study were masked to other aspects of the status of the participants	No	No	Yes	Yes	No	Yes	Yes	No	No	Yes	No	No	No
Describe any assessments undertaken for quality assurance purposes (e.g. test/retest of primary outcome measurements)	Yes	Yes	No	Yes	No	No	Yes	No	No	No	No	No	No
Explain any patient exclusions from analysis	Yes	No	No	Yes	No	Yes	Yes	Yes	No	Yes	Unclear	No	No
Describe how confounding was assessed and/or controlled	Yes	No	Yes	No	Yes	Yes	Yes	Yes	Yes	Yes	Yes	Yes	Yes
If applicable, explain how missing data were handled in the analysis	NA	NA	No	NA	Unclear	NA	NA	NA	NA	NA	Unclear	NA	NA
Summarize patient response rates and completeness of data collection	Yes	Yes	Yes	Yes	Yes	Yes	Yes	Yes	Yes	Yes	Yes	Yes	Yes
Clarify what follow-up, if any, was expected and the percentage of patients for which incomplete data or follow-up was obtained	Unclear	Unclear	No	No	No	No	No	No	No	No	No	No	No

NA: not applicable.

## Discussion

The current systematic review and meta-analysis showed that irrespective of the cut-off value of AHI(5 or 15 events/h), patients in the OSA group had higher rate of CAC presence and mean CAC score than those in the control group. These findings suggest that regardless of the cut-off value of AHI, OSA may be associated with risk of subclinical CAD. However, the pooled results in the current study must be interpreted cautiously due to lack of adjustment for confounding factors. The subgroup analyses showed that the OSA group had higher rates of CAC presence and mean CAC scores than the control group in patients monitored with HSAT or in-hospital/laboratory PSG, with the exception of the comparison of mean CAC scores using AHI cut-off of 5events/h in patients using HSAT, which was not significant. Pair-wise comparison showed that presence of CAC was associated with mild and moderate OSA but not with severe OSA. Mean CAC scores were significantly higher in patients with moderate and severe OSA than in patients without OSA, suggesting that CAC score increases with OSA severity. Since some analyses included a small number of studies (<3 studies), further studies are needed to confirm the results.

Although the present study showed that presence of OSA was associated with presence and extent of CAC, other cardiovascular risk factors such age, gender, BMI, diabetes, and hypertension were not considered in the meta-analysis. With regard to age, Arik *et al.* reported that age and AHI were independent predictors of CAC in patients with OSA [16]. The optimal cut-off values to predict CAC were age > 45 years and AHI >16 events/h with a sensitivity of 88.9% and 77.8% and a specificity of 54.3% and 56.5%, respectively, and the combination of AHI >16 and age >45 had increased specificity (70.6%) and unchanged sensitivity (87%) for predicting subclinical atherosclerosis [16]. Patient age in most of the studies included in the meta-analysis was greater than 45 years, except in Arik *et al.* [16] Kepez *et al.* [20] and Sorajja *et al.* [18], which may have been reflected in the wide error bar of calculated OR in the analysis of CAC presence in Arik *et al.* (Figure 2(B)). With regard to sex, Medeiros *et al.* demonstrated that there was an independent association with moderate to severe OSA (AHI ≥15 events/h) and the presence of CAC in middle-aged women [32]. Similarly, Weinreich *et al.* showed that the severity of OSA was independently associated CAC score in women of any age, whereas the association was only observed in men aged ≤65 years [17]. In contrast, Seo *et al.* recruited predominantly male (92.8%) subjects who were aged <65 years, and the results showed that AHI and moderate to severe OSA were not associated with the presence of CAC after adjustment for BMI [19]. A similar study conducted by Kim *et al.* recruited middle aged-men (40–49 years old) who were classified into AHI severity quartiles [21]. The results also showed that the association between AHI in the fourth quartile and CAC was no longer significant after further adjustment for BMI. The inconsistency of results from the Seo *et al.* [19] and Kim *et al.* [21] studies may be the result of enrolment of only Asian patients [36,37] and the lower prevalence of hypertension in these studies [38]. Moreover, with regard to BMI, it was reported that there was an independent association between BMI ≥23.0 kg/m^2^ and CAC regardless of OSA severity [21]. Similar results were also found among middle-aged men and women in a study published by Luyster *et al.*, which showed that among subjects with BMI ≥30 kg/m^2^, OSA severity was not associated with the presence of CAC [34]. In addition, Matthews *et al.* reported that such association became nonsignificant after adjusting for BMI in middle-aged men and women [35]. These findings suggest that obesity is a confounder of the association between OSA and subclinical CAD. Further research is warranted to confirm this finding and to identify other possible confounders.

Although the current study showed that patients with OSA had higher mean CAC score than those without OSA, CAC scores were presented differently among studies, including as mean ± SD, mean (IQR), median (IQR), or median (range), and the reported SD, IQR, or range were extremely large (Table 2). This indicates that the distribution of CAC score within each OSA severity group was heterogeneous. Moreover, calculation of mean and SD from mean and IQR, median and IQR, or median and range may result in deviation from the variance of the data, which could introduce bias in the pooling. In addition, the correlation of AHI and CAC score was uncertain. A weak (*r* = 0.342, *p* = .003) or even non-linear (*r* = 0.02, *p* = .90) correlation was found between AHI values and CAC score [16,31]. These results suggest that CAC may be more influenced by the duration of morbidity attributable to sleep apnoea rather than exhibiting a temporal increase in disease severity [31]. On the other hand, log-transformed AHI was independently associated with log-transformed CAC score. There was an association between doubling of AHI with an increase of CAC score in men aged ≤65 years (19%, 95% CI = −0.0008–42%) and in women of any age (17%, 95% CI = 3–33%) [17]. Although the pair-wise comparison in the current study partially showed that CAC score may increase with increased severity of OSA, significant association between CAC presence and severe OSA was not observed. A cluster analysis reporting on a very severe OSA phenotype based on AHI found that AHI had no direct link to cardiovascular diseases, and that for moderate-to-severe OSA phenotype, OSA severity was not associated with any comorbidities. The author speculated that the entity of nocturnal desaturation might have a stronger effect on the development of comorbidities than simply the frequency of apneas and hypopneas [39]. Further studies are warranted to examine the correlation between AHI and CAC score and the impact of additional variables on CAC.

In some of the included studies, HSAT instead of the gold standard PSG was used for diagnosis of OSA. Although HSAT has been validated with acceptable performance in identifying OSA, it may misestimate AHI [40,41,42]. A previous study reported that for PSG, an AHI cut-off of ≥5 events/h obtained the best receiver operating characteristic curve. For home respiratory polygraphy, an AHI cut-off of ≥10 events/h successfully confirmed the diagnosis [40]. The present study echoed the previous study showing that the presence of CAC was increased by a factor of 3.54 in in-hospital/laboratory PSG relative to a factor of 1.59 in HSAT for an AHI cut-off of ≥5 events/h. Moreover, mean CAC score was not significantly different in the comparison of AHI of ≥5 vs. <5events/h for HSAT. In addition, it was reported that AHI value for diagnosis of OSA using portable monitors ranged between 5 and 20 in different studies [43].

A previous systematic review evaluated the relationship between OSA and subclinical cardiovascular disease assessed by CAC, carotid intima-media thickness, flow-mediated dilation, and pulse wave velocity. In general, most of studies showed a correlation between markers of subclinical atherosclerosis and OSA severity. Of the six reviewed studies that used CAC to assess for subclinical atherosclerosis, four studies showed an independent correlation between AHI and CAC score, and two studies showed that the correlation became nonsignificant after adjusting for BMI or age [44]. Therefore, the findings of the previous review were consistent with those of the current study showing the positive association between the presence of OSA and subclinical CAD. Moreover, several previous meta-analyses have shown the association between OSA and subclinical CAD, asassessed by carotid intima-media thickness, flow-mediated dilation, and pulse wave velocity [45–47]. The present meta-analysis further confirmed the correlation through the assessment of CAC.

The present study has some limitations that should be considered. First, most of included studies were cross-sectional in design in which temporal or causal relationships between sleep apnoea and CAC presence cannot be determined and selection bias may occur. Second, patients with CAC scores of >100 were reported to have the greatest risk of coronary events [13]. The cut-off value for CAC presence was >0 in most of the eligible studies, and the association of low CAC scores and the risk of CAD may be questionable. Two eligible studies reported the outcomes of patients with a CAC score >100 [32,35] and one study reported the outcomes of patients with a CAC score >400 [33]. Although the study number was small, the pooled results show a glimpse that the OSA group had a higher rate of CAC presence for comparison of AHI ≥5 vs. <5 events/h for both CAC scores >100 and >400 (Table S1). Third, as mentioned above, CAC scores reported by the included studies were in different forms and their distribution indicated high heterogeneity. Therefore, the data may have been over-analyzed for pooling, which could result in bias. Fourth, the comparison groups in the current analysis were derived from combined data of different OSA severity groups, which could also result in data over-analysis. Further analysis such as meta-regression of the potential confounding factors was not conducted. Moreover, adjusted data reported in the included studies were not adjusted for identical confounding factors, making pooling impossible. Indeed, the best approach would be to conduct analyses using individual patient data. Lastly, the current study only considered AHI to quantify OSA severity. Several studies have reported that AHI failed to characterise the degree and duration of oxygen desaturation and cannot fully predict the symptoms and prognosis of sleep apnoea [39,48,49]. Therefore, other factors such as hypoxic burden can be introduced to better measure the severity of OSA.

In conclusion, this systematic review and meta-analysis found that the presence of CAC and CAC score may be associated with the presence of OSA. However, potential confounders such as age, gender, and BMI and the diversity of CAC scores may affect this association. Moreover, due to the limitations of the analysis of CAC score and OSA, it is recommended that other markers of subclinical CAD in addition to CAC score be considered in clinical practice when managing patients with OSA. It may be helpful for further research to explore these issues with individual patient-level meta-analysis.

## Supplementary Material

Supplemental Material

## Data Availability

The authors confirm that the data supporting the findings of this study are available within the article and its supplementary materials.
