# Supplementary material for: Association between apnea-hypopnea index and coronary artery calcification: a systematic review and meta-analysis
Source: Ann Med. 2021 Feb 1;53(1):302–17. doi: 10.1080/07853890.2021.1875137 (PMC7877988; doi:10.1080/07853890.2021.1875137)
Supplement: Supplemental Material [file IANN_A_1875137_SM1592.docx]

| **Comparison** | **Pooled statistics** | **N of studies** | **Heterogeneity test** |
| --- | --- | --- | --- |
| **CAC score > 100; CAC score > 400** |  |  |  |
| AHI ≧ 5 vs. < 5 | OR=2.73, 95%CI=(1.03, 7.23), p-value=0.043 | 3 | Q-value=11.507, df=2, p-value=0.003, I2=82.62% |
| AHI≧15 vs. < 15 | OR=2.53, 95%CI=0.58, 10.97), p-value=0.215 | 2 | Q-value=4.836, df=1, p-value=0.028, I2=79.32% |
| **CAC score > 100** |  |  |  |
| AHI ≧ 5 vs. < 5 | OR=4.19, 95%CI=(1.32, 13.35), p-value=0.015 | 2 | Q-value=3.225, df=1, p-value=0.073, I2=68.99% |
| AHI ≧ 15 vs. < 15 | OR=6.25, 95%CI=(1.66, 23.52), p-value=0.007 | 1 | Not assessed. |
| AHI, apnea-hypopnea index; CAC, coronary artery calcification; OR, odds ratio | | | |

**Table S1 Association of OSA severity and presence of CAC for CAC scores > 100 and > 400**
